# Temperature Insensitivity and Behavioural Reduction of the Physiological Stress Response to Longline Capture by the Gummy Shark, *Mustelus antarcticus*

**DOI:** 10.1371/journal.pone.0148829

**Published:** 2016-02-17

**Authors:** Leonardo Guida, Terence I. Walker, Richard D. Reina

**Affiliations:** School of Biological Sciences, Monash University, Clayton, Victoria, 3800, Australia; Centre for Cellular and Molecular Biology, INDIA

## Abstract

Many factors influence the physiological stress response to fisheries capture in elasmobranchs. However, the influence of sea surface temperatures (SST) and behaviour are unknown and crucial considering global fishing pressures. We investigated the effect of SST and behaviour on the physiological stress response to capture of the gummy shark, *Mustelus antarcticus*, and compared our results to a laboratory study using similar conditions to test whether stress responses of *in situ* capture are consistent with those from laboratory simulations. Capture time for 23 *M*. *antarcticus* ranged 32–241 min as measured by hook timers or time depth recorders (TDR) in SSTs ranging 12–20°C. TDR data from 13 *M*. *antarcticus* were analysed to quantify capture behaviour as the percentage of time spent moving during capture. Several physiological variables measured from blood samples obtained immediately upon the animals’ landing indicated that although warmer SSTs increased metabolic rate, the stress response to capture was not exacerbated by capture duration. During capture movement occurred for an average of 10% of the time and since *M*. *antarcticus* can respire whilst stationary, restricted movement probably mitigated potential influences of increased SSTs and capture duration on the stress response. Previous laboratory findings were also shown to be indicative of *in situ* conditions and we thus advise that studies control for water temperature given the influence it has on variables (e.g. lactate) used to measure capture stress in elasmobranchs. We highlight the importance of seasonal water temperatures and capture behaviour when assessing the resilience to fisheries capture and the implementation of appropriate fisheries management strategies.

## Introduction

Elasmobranchs are particularly vulnerable to fishing pressures, largely due to their low biological productivity [1] with one quarter of all species worldwide on the IUCN Red List of Threatened Species [2]. A critical challenge facing the conservation and sustainability of elasmobranch populations is the reduction of bycatch mortality [3], as elasmobranchs can constitute a significant portion of total bycatch [4] and are often discarded as a result of either fisheries regulations or low commercial-value [5].

The physiological stress response to capture in elasmobranchs, which is extensively described by Skomal and Mandelman [6], is complex and can vary depending on the fishing method [3], the duration of capture [7, 8] and respiratory mode of a given species [9]. The stress of capture can cause death or alterations to behaviour, growth and immunological function [10, 11] and the magnitude of stress experienced must be quantified in order to predict lethal and non-lethal consequences of capture.

Capture duration is a critical factor when determining the degree of stress experienced [12]. In longline fisheries, longer capture durations can increase mortality rates for some species such as the sandbar, *Carcharhinus plumbeus*, blacktip, *Carcharhinus limbatus*, and blacknose, *Carcharhinus acronotus*, sharks [13]. Conversely, it has also been shown that longer capture durations may assist physiological recovery from initial capture stress [7]. A major component of variation in the stress response, and ultimately mortality rates, to longline capture is thought to be associated with metabolic scope [14].

Seasonal water temperatures can influence the metabolic rate of elasmobranchs and their physiological stress response to capture. Most chondrichthyan species are ectotherms and have their metabolic rate influenced by the surrounding water temperatures [15]. As water temperature (and metabolic rate) increases, the physiological stress experienced may become greater, reducing resilience to capture and increasing mortality rates [16, 17]. Seasonal stress responses to capture have been documented in the little skate, *Leucoraja erinacea*, [18] and Atlantic sharpnose shark, *Rhizoprionodon terraenovae*, [19] but no study as yet has examined how variation in water temperature may exacerbate or ameliorate the physiological response during capture. If the effects of water temperature are understood, it may be feasible to reduce mortality rates by seasonal closures and other restrictions on the use of specific fishing gears, methods and practices.

Further understanding of the stress response can be gained by examining the behavioural response to a stressor. Using established physiological stress markers, behavioural correlates can be identified and assist in characterising the response of a species to environmental stressors, such as housing stress in dogs [20], transport stress in sheep [21] and habitat selection in bears [22]. Correlations between quantified behaviour and the physiological response to capture in elasmobranchs have thus far been limited to post-capture events [23], and as yet behaviour during capture in the wild has not been quantified. Investigating capture behaviour in elasmobranchs can provide a better understanding of both the physiological stress response and the mortality risk relative to particular fishing methods.

Capture events in the wild are difficult to observe and descriptions of how capture behaviour relates to measured stress variables are largely qualitative; inferred from *in situ* observations and the species’ metabolic scope [7, 24, 25]. Quantified capture behaviour and its influence on the stress response has currently been limited to the laboratory study of Frick et al. [26], which related blood biochemistry to the struggling profile of Port Jackson shark, *Heterodontus portjacksonii*. As yet there are no quantitative assessments of capture behaviour *in situ* and how this relates to the measured stress response. Time depth recorders (TDR) offer a useful opportunity to measure capture behaviour in longline fisheries. TDRs can be individually placed near hooks and measure depth changes associated with movement of the animal during capture. This approach has only been applied in one study, which measured the capture duration and resurfacing behaviour of a loggerhead turtle, *Caretta caretta*, caught on a longline [27]. Quantifying the behaviour of captured elasmobranchs may explain variability in measured stress variables and characterize species-specific responses to longline capture.

*In situ* studies of capture stress are limited in their ability to assess the post-capture fate of released elasmobranchs. Limitations can include both the expense of tag-tracking technology [23] and the extremely limited ability to reassess the physiological condition of the animal after release. In contrast, laboratory studies offer controlled conditions and the ability to monitor the post-capture physiological recovery or lack thereof with relative ease [8, 11]. However, due to potentially confounding factors such as transport and captivity stress prior to and during laboratory treatments [28], whether laboratory results of post-capture physiology can be accurately representative of and extrapolated to *in situ* captures is yet to be determined. Given that costs and logistical difficulty are higher for *in situ* than for laboratory studies, there is a need to resolve the uncertainty in results from laboratory studies associated with these confounding factors.

Our study had two main aims; 1) to investigate the effect of water temperature on the physiological stress response to capture of known durations, and 2) to compare the *in situ* physiological assessment of capture stress with that from the laboratory based study of Frick et al. [8]. To address these aims, we characterised the physiological stress response of the gummy shark, *Mustelus antarcticus*, to capture on demersal longlines and also employed a novel method using TDRs to quantify the capture behaviour. *Mustelus antarcticus* is Australia’s most valuable commercial shark species, is targeted with demersal gillnets, retained as byproduct from other fishing methods and discarded as bycatch (due to commercial quota and minimum length limits) in various commercial and recreational fisheries. Assessment of their tolerance to longline capture in the wild is of importance to achieve the sustainable use of this species. We hypothesized that although increased water temperatures would increase metabolic rate and hence the magnitude of the physiological stress response, restricted movement during capture (i.e. the ability to respire whilst stationary) may mitigate the potentially exacerbating effects of temperature during capture.

## Methods

Our study was conducted between March 2012 and December 2013 in Western Port bay, Victoria, Australia (38.433° S, 145.376° E). This study was approved by and performed in accordance to Monash University Animal Ethics Committee approval BSCI/2009/16 and Fisheries Victoria permits RP1000 and RP1115.

### Collection of M. antarcticus

Twenty-three male (n = 12) and female (n = 11) *M*. *antarcticus* were caught on a commercially-operated vessel using a demersal longline (mainline length: ~2 km, set depth: 3.5–22.7 m) with an average soak-time (end of line deployment to the end of line retrieval) of 270 (± 15 S.E.; all means are henceforth reported with standard error) min. Depth recordings using TDRs (LAT1100, Lotek Wireless, Newfoundland, Canada) matched bathymetric data (GPSMAP 555, Garmin, USA) confirming that the mainline of each set was weighted to sit on the sea floor. Each of the 500 hooks (Milward Superbaiter 7/0 hooks) was attached to a braided polyethylene gangion (the line connecting the hook to the mainline) measuring 70 cm in length. Gangions were spaced ~6 m apart. Fifty gangions were attached to hook timers (resolution 1 min, Lindgren Pitman HT600 Hook Timer, Pompano Beach, Florida, USA) and connected to the mainline, while another 40 hooks had TDRs fixed to the gangion at 10 cm from the eye of the hook to avoid losing TDRs to bite-offs. Due to a methodological oversight, TDRs did not initially record temperature and SSTs on the day of capture were recorded using an on-board fish-finder with temperature sensor (Lowrance X52, Lowrance, USA). Separate temperature recordings of our fishing locations in Western Port bay recorded an absolute temperature difference of 0.1°C between the on-board sensor and TDRs placed at the surface, and an absolute difference of 0.3°C between 0 and 11.5 m depths. Due to logistical difficulties, we were not able to record at our max depth of 22.7 m. Nonetheless, we are confident that SSTs in our study reflect bottom temperatures in the water column (where sharks were caught) because of the fast tidal flows which provide a thorough mixing of surface and bottom waters in Western Port bay where longlines were set [29].

### Sampling procedure

*Mustelus antarcticus* were landed on deck individually, immediately restrained, measured for total length (TL) and had up to 2 ml of blood sampled by caudal venipuncture in prepared heparinised syringes. Blood sampling took less than 30 sec and occurred within 1 min of landing the animal. For animals caught on hooks with hook timers we recorded the elapsed time since the timer was triggered. After sampling, animals were either immediately euthanised by decapitation (if retained for commercial sale), retained and transported to aquaria (if required for additional experimentation) or released.

### Blood analysis

Immediately after obtaining a sample, whole blood was analysed for pH and lactate in a portable iStat blood gas analyser (CG4+ cartridge, Abbott Laboratories, Chicago, USA) thermoset to 37°C. The iStat provides an accurate measurement of *M*. *antarcticus* lactate concentrations [30] although it is limited to reporting concentrations below 20 mmol L^-1^ and so concentrations exceeding this limit were later analysed in the laboratory as described below. Values of *p*CO_2_ measured by the iStat are not reported in our study as it has recently been demonstrated in sharks that correcting *p*CO_2_ values across a range of temperatures is not accurate [31]. The remaining whole blood was kept on ice until returning to the laboratory for continued analysis. Haematocrit was determined by drawing ~70 μm of whole blood into microcapillary tubes and centrifuging at 10,000 rpm (Hawksley Haematospin 1400, Sussex, UK) for 5 min. The remaining whole blood sample was also centrifuged at 10,000 rpm for 5 min and had the plasma portion removed for analyses of lactate, glucose, potassium and osmolality. Plasma lactate and glucose concentrations were measured in the laboratory with a Radiometer ABL800 Basic (ABL; Radiometer Medical, Denmark). Osmolality (mmol kg^-1^) was measured using an Osmomat 030 (Gonotec, Berlin, Germany). Potassium concentrations (mmol L^-1^) were determined by an external pathology laboratory using a Beckman Synchron LX20PRO Analyser (Beckman Coulter, Fullerton, California, USA).

Values obtained for pH using the iStat were temperature-corrected and although such conversions are ideally species-specific [32], there are currently no data for *M*. *antarcticus*. Therefore, conversions were as described in Harter et al. [31] to give *in vivo* values.

### Quantifying capture duration and behaviour

**Fig 1:**
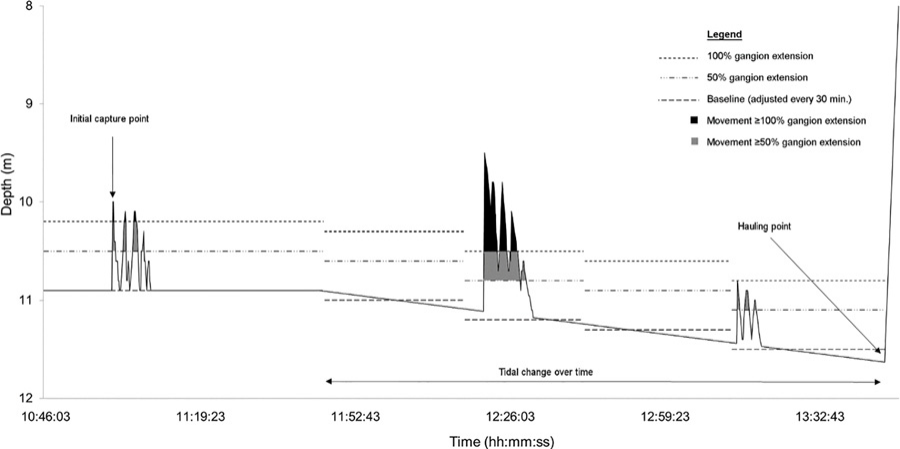
Stylised capture behaviour profile from a time depth recorder (TDR) of *Mustelus antarcticus* on demersal longline. Shifts in the calculated baseline incorporate tidal depth changes for every 30 min. Movement is considered to have occurred if the depth profile equals or exceeds (at a minimum) the threshold of 50% gangion extension.

Hook timers (n = 10) and TDRs (n = 13) were used to calculate the time spent on the hook for a subset of animals. TDRs recorded the time and depth of initial capture with a depth resolution and accuracy 0.05% and 1%, respectively. Vertical movements in the water column were used to determine capture behaviour. TDRs were programmed using TagTalk (Lotek Wireless, Newfoundland, Canada) to measure depth every 2 sec. A sampling interval of 2 sec was chosen to provide maximum resolution of depth change within tag memory capacity over several hours. The capture point (identified by an initial marked depth change) and the total time (min) spent moving whilst captured were quantified as total deviations from the baseline depth profile. The deviations which were considered movement of the captured animal were equal to or above the threshold of 100% gangion extension (~60 cm) or 50% gangion extension (~30 cm), respectively, indicating either an attempt to escape or intermediate movement whilst captured. Based on the mean TL of 778 (± 15) mm from *M*. *antarcticus* caught on TDRs, these thresholds also indicate that at a minimum, ‘intermediate’ movement equated to a distance of at least 39% of the animal’s TL. Thus, an individual’s total movement was categorised using both degrees of sensitivity. The method for identifying capture points, quantifying capture duration and total movement on TDRs is described in five steps below and is also illustrated in a stylised TDR trace (Fig 1);

1. To detect initial capture point and subsequent struggle/movement periods, a baseline was calculated by averaging a steady-state depth over a 1-min period immediately before the initial capture point (as validated by step 4). Since tide could influence recorded depth, baselines were recalculated (also over 1min periods) at the end of every 30 minutes. In the event movement periods occurred over a significant tidal depth change, the baseline was recalculated at this period by averaging the depths at 1 min before and 1 min after the respective movement period.
2. The absolute difference between actual recorded and baseline depth was calculated at each data point so that movement could be identified and quantified.
3. To create the thresholds at which animal movement could be inferred, the respective gangion lengths were summed with the standard deviation (s.d.) of the baseline to account for natural depth change influenced by surface swell.
  - Threshold at 100% extension = 0.60 m + s.d.
  - Threshold at 50% extension = 0.30 m + s.d.
4. Where threshold values were met or exceeded by the absolute difference in depth (step 2), movement was confirmed. Movement was deemed to have ceased if the threshold was not met or exceeded for more than 4 sec. This was a conservative estimate based on observations of *M*. *antarcticus* on simulated longline capture in aquaria (L. Guida *pers*. *obs*.).
5. Points beyond the point of hauling gear up (visually determined and checked with time of animal landing) were not included in the analysis.

It was not possible to determine the intensity of movement as fast/vigorous or slow during capture because swimming can occur at a constant depth above the sea floor for a period of time (indicated by a flat line).

### Statistical analysis

To determine whether capture durations calculated from TDR data differed significantly from capture durations detected by hook-timers, we used a Bayesian estimation to explore the differences between the two methods. To investigate the influence of threshold sensitivity on the percentage of time where movement was recorded during capture (henceforth referred to as ‘total percentage movement’), a Bayesian estimation was used to explore the differences between the two methods.

Initial exploratory analysis used six independent generalized additive models (GAM), one for each blood variable (response variable), to test for non-linear relationships between each blood variable and both the capture duration and SST (predictor variables). Since non-linear models were not better fits than linear models, each blood variable was plotted individually as a response in Bayesian multiple linear regression models. No models indicated significant interactions between SST and capture duration and thus all models were subsequently additive regressions. In all models lactate was log transformed to meet assumptions of normality. Only one individual was landed dead and was subsequently removed from the analysis because moribund *M*. *antarcticus* have significantly altered blood biochemistry compared with live individuals [8].

Bayesian statistics were employed as they are not overly restricted by sample size [33]. In all Bayesian models, diagnostic matrix plots were used to test for autocorrelation of variables and ensure model performance. Models included a non-informative prior distribution mean of 1 x 10^−6^ and a Markov Chain Monte Carlo estimation (iterations = 5 x 10^−4^, burn-in = 2000, thinning = 10). Significance was determined when values reported in the credibility intervals (CI) between 2.5 and 97.5% did not cross or include the value of 0. All analyses were performed using R v.3.1.2 [34].

To assess differences between laboratory and field-based studies, results from our study were compared to those of *M*. *antarcticus* reported by Frick et al. [8].

## Results

A total of 23 *M*. *antarcticus* with known capture durations were caught in SSTs ranging from 12–20°C. Total length ranged from 560–1628 mm with a mean of 927 ± 62 mm. Based on TDR data, all animals were caught at less than 11.0 m with the exception of one animal caught at 20.9 m. The average depth of capture was 7.9 ± 0.3 m.

Total capture duration calculations from TDRs were negligibly influenced using either of the thresholds chosen as the detection of initial capture points for any given individual differed by up to 23 sec and at an average of 7 ± 0.8 sec. Thus, the 50% threshold was arbitrarily selected to calculate capture durations from TDRs.

**Fig 2:**
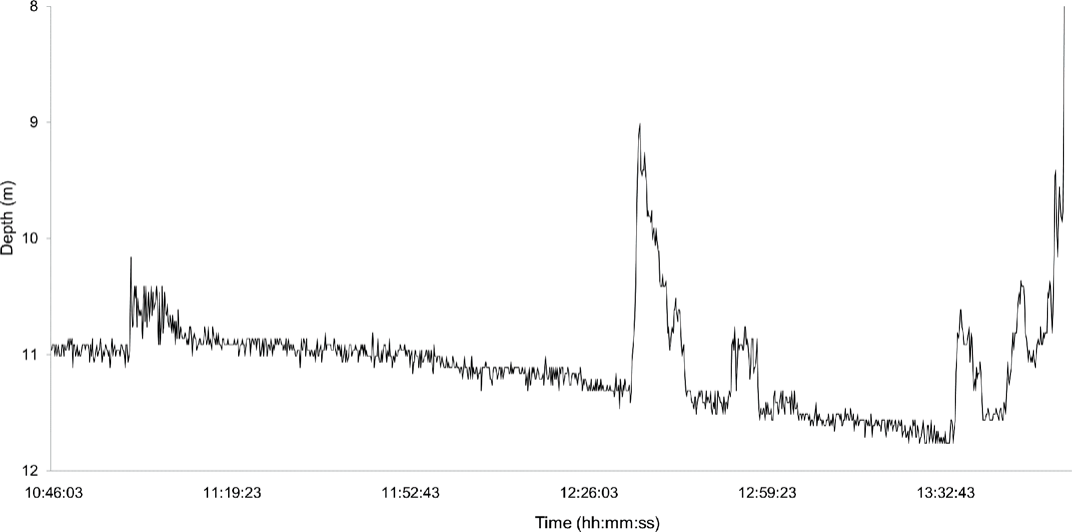
Real capture behaviour profile from a time depth recorder (TDR) of *Mustelus antarcticus* on demersal longline. Using our quantification method, capture lasted 174 min with a total of 16% and 8% total movement at the respective thresholds of 50% and 100% gangion extension.

Capture durations measured from TDR data were not significantly different to capture durations recorded on hook timers (μ = -0.201, CI_2.5, 97.5_ = -52.285, 52.725) confirming that our method of detecting the initial capture point was accurate. Total percentage movement during capture was significantly greater using the 50% than the 100% threshold (μ = -1.032, CI_2.5, 97.5_ = -1.528, -0.550). Fig 2 is an example of an actual TDR trace of a captured *M*. *antarcticus* with calculated capture duration and total percentage movement. Descriptive statistics regarding means and ranges of capture duration and percentage movement obtained from hook timers and TDRs are listed in Table 1.

**Table 1:** Descriptive statistics of capture duration (min.) and total movement obtained from *Mustelus antarcticus* caught on time depth recorders (TDR) and hook timers.

Measuring device		Mean (± standard error)	Range
TDR (n = 13)			
	Total capture duration (min)	160.9 ± 14.1	31.7–219.7
	*50% threshold*		
	Movement (min)	17.3 ± 2.0	0.5–76.0
	Movement (% time)	10.4 ± 1.2	0.3–50.3
	*100% threshold*		
	Movement (min)	6.8 ± 1.0	0.0–40.0
	Movement (% time)	4.0 ± 2.1	0.0–22.4
Hook timer (n = 9^a^)			
	Total capture duration (min)	154.9 ± 22.1	73.0–241.0
TDR + hook timer			
	Total capture duration (min)	158.1 ± 2.6	31.7–241.0

^a^ one individual was removed from all analyses as it was landed dead. See ‘Statistical analysis‘ in Methods.

**Fig 3:**
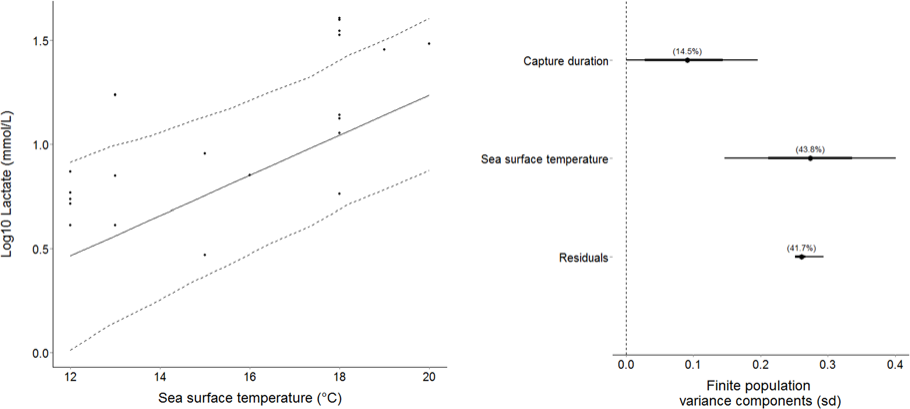
Increased plasma lactate (log10) as a result of increased sea surface temperature (left). Dashed lines are representative of 95% credibility intervals. **The percentage variance in plasma lactate explained by capture duration and sea surface temperature (right)**.

**Fig 4:**
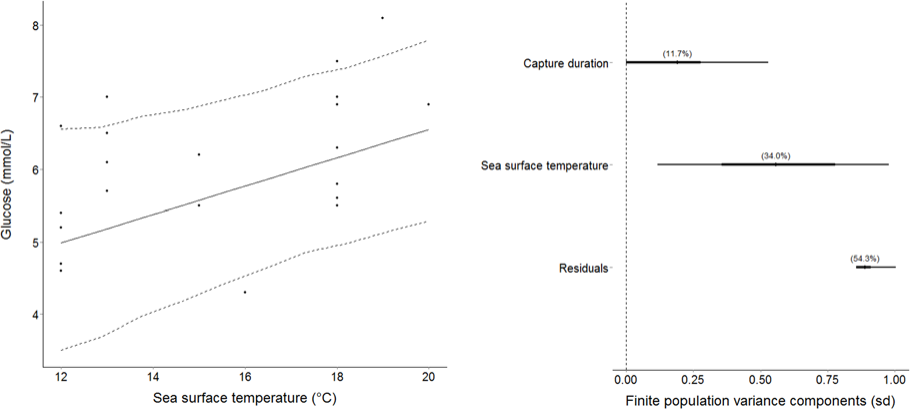
Increased plasma glucose as a result of increased sea surface temperature (left). Dashed lines are representative of 95% credibility intervals. **The percentage variance in plasma glucose explained by capture duration and sea surface temperature (right).**

Capture durations did not significantly affect any measured blood variables (Table 2). SST significantly affected several blood variables (Tables 2 and 3); increases in SST were associated with increased lactate (Tables 2 and 3; Fig 3) and glucose (Tables 2 and 3; Fig 4). Conversely, K+ (Tables 2 and 3) and osmolality (Tables 2 and 3) decreased as temperature increased. Haematocrit and pH were not significantly affected by SST (Tables 2 and 3).

**Table 2:** Bayesian additive linear regression estimates of *Mustelus antarcticus* blood variables (response) predicted by capture duration and sea surface temperature (SST).

Response variable	Parameter estimates for each predictor variable					
	Capture duration			Sea surface temperature		
	μ ± s.d.	CI 2.5%	CI 97.5%	μ ± s.d.	CI 2.5%	CI 97.5%
**pH**	0.000 (0.001)	-0.002	0.001	-0.027 (0.015)	-0.057	0.004
**Lactate**	0.004 (0.003)	-0.002	0.009	0.221 (0.53)*	0.118*	0.325*
**Glucose**	0.003 (0.004)	-0.005	0.011	0.196 (0.078)*	0.041*	0.349*
**Potassium**	0.002 (0.003)	-0.004	0.008	-0.163 (0.063)*	-0.289*	-0.038*
**Osmolality**	-0.001 (0.000)	-0.001	0.000	-0.013 (0.006)*	-0.026*	-0.001*
**Haematocrit**	-0.020 (0.015)	-0.050	0.009	-0.546 (0.299)	-1.135	0.352

Values reported are the mean increase or decrease (μ) ± standard deviation (s.d.) in the variable examined per capture duration increase of 1 min or SST increase of 1°C. Credibility intervals (CI) are between 2.5 and 97.5%.

* Significant values whereby CI do not cross the value of 0.

**Table 3:** Predicted means (μ) of blood variables across specific sea surface temperatures (SST), as derived from Bayesian regression estimates.

Blood variable	Parameter estimates of each predictor variable for each of the three values of SST (°C)								
	12.0			16.4			20.0		
	μ	CI 2.5%	CI 97.5%	μ	CI 2.5%	CI 97.5%	μ	CI 2.5%	CI 97.5%
**pH**	7.424	7.123	7.733	7.306	7.058	7.552	7.212	6.690	7.461
**Lactate (mmol L**^**-1**^**)**	2.938*	1.050*	8.147*	7.852*	3.459*	18.408*	17.258*	7.396*	39.719*
**Glucose (mmol L**^**-1**^**)**	4.986*	3.453*	6.526*	5.855*	4.607*	7.097*	6.550*	5.340*	7.834*
**Potassium (mmol L**^**-1**^**)**	3.431*	2.165*	4.661*	2.709*	1.723*	3.729*	2.131*	1.128*	3.151*
**Osmolality (mmol kg**^**-1**^**)**	1.145*	1.025*	1.271*	1.086*	0.988*	1.199*	1.039*	0.937*	1.139*
**Haematocrit (% packed cell volume)**	27.324	21.342	33.041	24.897	20.232	29.759	22.955	18.132	27.762

Since capture duration has no significant effect on all blood variables, the values obtained are standardized at a capture duration of 0 min.

* Significant values whereby CI do not cross the value of 0.

## Discussion

The aim of this study was to determine how temperature and behaviour during capture influenced the physiological stress response of *M*. *antarcticus* when caught on a demersal longline. Our results indicate that *M*. *antarcticus* is relatively resilient to such capture for at least four hours and although increased SSTs are likely to increase the metabolic rate and anaerobic activity of white muscle in *M*. *antarcticus*, the physiological responses to capture were not affected by capture duration. Resilience to disruption of physiological stress response during capture is thought to be attributed to a combination of reduced metabolic scope and increased respiratory efficiency during capture [14, 35, 36]. In our study we report the behaviour of *M*. *antarcticus* during capture and think that minimal movement during capture and the ability to respire whilst stationary mitigated the stress response particularly at higher SSTs when the metabolic rate was assumed to be higher. The results of our study are comparable to Frick et al. [8], who used similar capture durations under a similar temperature range, which suggest that results from laboratory studies are reasonably indicative of those *in situ*.

The metabolism of ectotherms is dependent on the surrounding temperature and thus an increase in metabolic rate in *M*. *antarcticus* could account for increases in lactate and glucose at higher SSTs. At similar temperature ranges to our study, the metabolic rate of the bat ray, *Myliobatis californica*, increased three fold as water temperature rose from 14 to 20°C [15]. However, despite the influence of temperature on metabolic rate and changes in variables associated with capture stress, the stress response to capture was not exacerbated across varying capture durations.

We think the capture behaviour exhibited by *M*. *antarcticus* mitigated increases in the stress response to capture. As quantified by TDRs, we were able to show that movement occurred for an average of 5–10% of the total time captured, with the animals probably resting on the seafloor when not moving. The ability to respire whilst stationary and the minimal movement observed during capture are important factors explaining why the physiological stress response to capture was unaffected by duration despite an increased metabolic rate at higher temperatures. The ability to respire whilst movement is restrained is a key factor contributing to mortality across fishing gear types [9]. In both Brooks et al. [7] and Gallagher et al. [35] the attenuation of the stress response and increased resilience throughout capture was suggested to be related to behavioural responses and increased movement range to assist respiration. In our study, minimal accumulation of lactate and no significant decline in blood pH, irrespective of capture duration and activity level, not only suggest that there was minimal movement during capture but that sufficient oxygenation may have limited the contribution of CO_2_ to respiratory acidosis [37]. The ability to efficiently respire despite the considerably restricted movement range of up to ~90% mean TL (gangion length = 70 cm) is further evidence to support the notion that *M*. *antarcticus* minimised its activity by remaining stationary on the sea floor and freely respired adjacent to the mainline.

By characterizing behavioural responses of several species it may be possible to determine the influence that gear configuration has on the stress response to capture. Additional TDR data from an array of species can show that proximity of the mainline to the sea floor may improve resilience to capture for species capable of stationary respiration. From our results we hypothesise that for stationary respiring species, demersal longlines are potentially less stressful than longlines set at the surface or mid-water. Low (15%) mortality rates of bull sharks, *Carcharhinus leucas*, in bottom-set longline captures [13] are hypothesised to be a result of the ability to respire whilst stationary [25], a behaviour that could feasibly be tested by application of TDRs. The depth setting of hooks has also been found to affect mortality rates in chondrichthyans as a result of water temperature changing with depth [17]. In our study, we are confident of a negligible change (< 1°C) in temperature in the water column because of thorough mixing of surface and bottom waters in Western Port bay [29]. However, in open oceans the temperature gradient at or beyond a similar depth to our study may be more prominent [14]. Increased struggling effort during capture may also be inversely related to gangion length. For ram-ventilating species, gangion length can limit movement and compromise respiratory potential [17]. TDRs can potentially reveal unique behaviours associated with gangion length whereby smooth, constant swimming or erratic depth changes may be associated with the degree of restraint on the line. Furthermore, given the negative buoyancy of chondrichthyans, for ram-ventilating species landed dead, the point of mortality during capture may also be identified by TDRs as a constant, flat-line at maximum depth. However, identifying points of mortality for stationary respirers may not be possible. Accelerometers can allow some estimations of the intensity and direction of movement and are potentially more informative than TDRs, but their significantly higher cost relative to TDRs may limit their application. Nonetheless, the use of accelerometers in the future should be investigated where possible.

The physiological stress response to capture of *M*. *antarcticus* is comparable between laboratory and *in situ* studies under similar conditions. The laboratory study of Frick et al. [8] examined longline caught *M*. *antarcticus* of a similar size (TL = 860 ± 10 mm) across a similar range of durations (30–360 min) and temperatures (11–22.8°C) to our study and also presented similar physiological profiles. In both studies, capture duration had no effect on lactate, glucose, potassium and haematocrit immediately following capture. The reported means for these variables are comparable between studies when comparing the grand mean capture duration of 160 min in our study with both the ‘commercial’ and 120 min durations presented in Frick et al. [8]. Notably, one individual in our study was landed dead with a potassium concentration (6.2 mmol L^-1^) similar to moribund sharks removed from capture (~6 mmol L^-1^) in Frick et al. [8].

Although the assessment of capture behaviour in Frick et al. [8] revealed that *M*. *antarcticus* rarely rested on the tank bottom, the gangion in Frick et al. (7) was considerably longer (150 cm) than ours (70 cm) and was suspended from above the tank providing ample movement range but a limited option to rest on the bottom (centre of the tank only) without placing excessive tension. Nonetheless, Frick et al. (7) noted there was no tension on the gangion throughout capture and similar physiological profiles to our study would suggest that in both studies, exertion throughout capture was minimal. Presumably little or no tension on the gangion in our study was achieved by resting on the sea floor or performing horizontal movements around the mainline. Interestingly, more recent laboratory observations reportedly show *M*. *antarcticus* to rest on the bottom of the tank for the majority of the time during capture providing the gangion itself is bottom-set (L. Guida *pers*. *obs*.). Despite slight variations in behaviour, the physiological stress recorded under similar capture conditions indicates that laboratory studies are reasonably accurate and viable options to simulate commercial capture, provided the animals are not affected by captivity and that the experimental setup is reasonably representative of commercial gear types and techniques. Furthermore, given that lactate is considered a good measure of capture stress in elasmobranchs [6], our results also indicate the importance of controlling water temperature’s influence when testing for the effects of factors such as gear type and fishing duration.

Based on the recovery and post-capture mortality estimates (up to 72 hours following removal from the gear) provided by Frick et al. [8], we expected post-release mortality from demersal longline capture to be low. For the purpose of a separate study, several captured *M*. *antarcticus* used in our study were retained alive in aquaria and experienced no mortality for up to seven days. Although the physiological response is comparable between *in situ* and laboratory caught *M*. *antarcticus*, further work is needed to determine the response of other species with respect to gear type.

## Conclusion

The incorporation of behavioural measurements during capture provides important information about the relative sensitivity of species’ tolerance to capture. As demonstrated by *M*. *antarcticus*, resilience to demersal longline capture is related to both its behavioural response during capture and the ability to respire whilst stationary. We suggest future studies employ TDRs in conjunction with other technologies, such as accelerometers and cameras, which permit the detection of behaviour to better understand capture behaviour and the importance of gear configuration.

Controlled laboratory studies can reasonably represent physiological responses to capture *in situ*. Thus, laboratory studies are viable options, from both a scientific and logistical standpoint, to further assess the stress response and tolerance of elasmobranch species to fisheries capture. By identifying species resilient to fisheries capture, it is possible to identify particular fishing practices conducive to sustainability and conservation of the respective species’ populations. Furthermore, by noting the influence of temperature on stress physiology, it is also possible to assess how seasonal and regional (depth and location of gear) effects may influence the resilience of a species whether such factors need to be considered when regulating the operation of longline fisheries.

We acknowledge that our study is based on a relatively small sample size and displays considerable variation among individuals; an issue typical of physiological studies on elasmobranchs [7]. However, as stated previously, the implementation of a Bayesian framework can mitigate such issues given that sample size imposes relatively little restriction on analysis. Nonetheless, future research directed at this topic using greater sample sizes should be conducted when possible.
